# Dosimetric verification of inverse planned step and shoot multileaf collimator fields from a commercial treatment planning system

**DOI:** 10.1120/jacmp.v3i2.2580

**Published:** 2002-03-01

**Authors:** M. A. MacKenzie, M. Lachaine, B. Murray, B. G. Fallone, D. Robinson, G. C. Field

**Affiliations:** ^1^ Department of Medical Physics Cross Cancer Institute 11560 University Ave. Edmonton Alberta Canada T6GIZ2

**Keywords:** IMRT, inverse planning, treatment verification, DMLC

## Abstract

An inverse treatment planning (ITP) module on a commercial treatment planning system (TPS) (Helax AB, Uppsala, Sweden) is being used for an in‐house clinical trial for treatment of nasopharyngeal cancer with contralateral parotid sparing. Intensity modulated radiation therapy (IMRT) fields are delivered by step and shoot multileaf collimator (MLC) with a DMLC enabled Varian 2300 CD (Varian Associates, Palo Alto, CA). A series of testing procedures have been devised to quantify the modeling and delivery accuracy of routine clinical inverse planned IMRT using Helax TMS and the Varian step and shoot MLC delivery option. Testing was done on specific aspects of the TPS modeling germane to DMLC. Measured relative dose factors (head scatter plus phantom scatter) for small MLC fields, normalized to a $10\times 10{\text{cm}}^{2}$ non‐MLC field, were found to differ by 2–3% from the TPS values for the smallest of the fields tested. Relative distributions for small off axis fields were found to be in good agreement. A process for the routine clinical verification of IMRT fields has been implemented. Each IMRT field in an inverse plan is imported into a flat water tank plan and a “beam's eye view” (BEV) dose distribution is generated. This is compared to the corresponding measured BEV dose distribution. The IMRT verification process has also been performed using an anthropomorphic phantom. Large clinical fields (i.e., greater than 14.5 cm in the leaf direction) caused difficulties due to a vendor specific machine restriction, and several techniques for dealing with these were examined. These techniques were (i) the use of static stepping of closed junctions, (ii) the use of two separate IMRT fields for a given gantry angle, and (iii) restricting the overall maximum field size used. The overall process has allowed implementation of an in‐house protocol for IMRT use on an initial clinical site. Results of the verification measurements for the first ten patients treated at this center reveal an average maximum dose per IMRT field delivered of 71.0 cGy, with a mean local deviation from the planned dose of – 1.2 cGy, and a standard deviation of 2.4 cGy.

PACS number(s): 87.53.Dq, 87.53.Tf

## INTRODUCTION

One of the goals of external beam radiotherapy is the delivery of a homogeneous tumourcidal dose to a planning treatment volume (PTV) while avoiding organs at risk (OARs). The optimal realization of such distributions requires fields with nonuniform energy fluence distributions. The use of such fields is referred to as intensity modulated radiation therapy (IMRT). It is anticipated that improved dose adherence to the PTV will lead to greater local control,^1^ ^,^ ^2^ and there is an emerging indication of reduced morbidity.^3^ ^–^ ^5^ IMRT delivery techniques include static compensators, step and shoot or sliding window multileaf collimators, and tomotherapy.

A number of studies have recently been published on IMRT QA, but most focus on experience with a commercially available serial tomotherapy device and its associated TPS.^6^ ^–^ ^10^ The planning and verification of IMRT fields is often complicated by the presence of high dose gradients in the field,^6^ in contrast to standard open fields. While step and shoot IMRT deliveries do not have to contend with the same junctioning issues of serial tomotherapy^11^ (i.e., the junctioning of adjacent transverse dose slices), both must contend with the delivery of small fields. Thus, verification of relative dose factors (head scatter, phantom scatter) and relative dose distributions associated with small MLC fields is essential.

A per patient verification procedure is desirable for the introduction of IMRT treatments into clinical practice.^12^ A number of issues are addressed, including closed leaf leakage, the number of levels and segments used, and the modeling of small fields by the TPS.

This center used Helax‐TMS to inverse plan a patient's clinical treatment on April 4, 2000. Results are presented for the first ten IMRT patients treated at this center. An in‐house protocol for nasopharynx (Phase 1: 50 Gy median PTV dose, Phase 2: 16 Gy median PTV dose, <20 Gy to 50% of parotid; stimulated and unstimulated salivary flow measured) is currently underway. A similar RTOG protocol for use of IMRT for head and neck treatment is under development.^13^ It should be noted that, while this work constitutes the authors' experience with IMRT and ITP with a particular TPS and linac combination, it is believed that there are a number of wider IMRT issues addressed that may of interest to a wider audience.

## MATERIALS AND METHODS

The delivery verification techniques examined in this paper are of two general types. The first are those used in the examination of the TPS MLC and DMLC modeling. The second are those used in the evaluation of planned field deliveries. The latter methods were used to evaluate both the deliveries on an anthropomorphic phantom, as well as for clinical cases.

Helax TMS employs a convolution/superposition dose calculation algorithm, the details of which are extensively discussed in the literature.^14^ ^–^ ^18^ The inverse planning module requires the user to set dose and volume constraints for target volumes and organs at risk. Plans are based on a fixed number of energy fluence levels, which was initially set to ten. The inverse treatment planning (ITP) module employs a gradient search algorithm^19^ on a random sampling of dose points in the volumes of interest, attempting to satisfy the given constraints. The algorithm stops when one of three conditions is met: (1) a solution is found, (2) the maximum time is exceeded, or (3) a maximum number of iterations is reached. When an acceptable plan is achieved and approved by the oncologist, the result may be exported as either an array of relative energy fluence values (called a modulation matrix) or a multileaf modulation (MLM) file. The former may be delivered using either physical compensators or dynamic MLC. The latter is a prescription for step and shoot MLC positions. This institute has opted for the use of the MLM file in the delivery of IMRT fields.

Testing MLC modeling consisted of measuring field distributions and relative dose factors for small fields, both on and off the CAX. Measurements were performed with film and ion chamber. A small volume (2 mm radius active volume) ion chamber was positioned in a dosimetry system water tank (Wellhöfer Dosimetrie, Nörnberg, Germany) and a series of test field distributions were scanned. An ion chamber was also placed on the CAX at the reference depth of 10 cm for measurement of relative dose factors for a series of fields. All relative dose factor measurements were normalized with respect to a reference field size of $10\times 10{\text{cm}}^{2}$ at a reference depth of 10 cm.

A series of films were placed in a Solid Water™ (Gammex RMI, Middleton, WI) phantom and exposed to a range of calibration exposures. Sets of test fields were then exposed to provide both relative OD and field distribution measurements. Using the same set of test fields, relative dose factor values and dose distributions were then calculated on the TPS for comparison to measured values.

**Figure 1:**
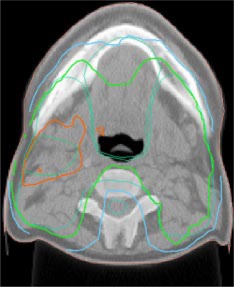
(Color) An initial conformal IMRT dose distribution with sparing of the contralateral parotid in an anthropomorphic phantom test plan.

Inverse IMRT planning was initially performed on an anthropomorphic phantom (Rando, Alderson Radiation Therapy). This anthropomorphic phantom was scanned on a CT simulator (AcQSim, Philips Medical Systems) and an IMRT plan was generated using the acquired CT data set. The inverse IMRT plan simulated a nasopharynx‐like case, as this was the first intended clinical site to be treated in accordance with an in‐house protocol. The anthropomorphic test treatment consisted of seven coplanar 6 MV beams designed to give a median 50 cGy to the PTV and constraints were specified in order to achieve contralateral parotid sparing (see Fig. 1). The planning process for this mock treatment closely resembled the planning flow currently employed clinically.

A Varian Clinac 2300 CD and 2300EX with dynamic dose software and MLC were used for IMRT delivery. A 52 leaf MLC, consisting of a set of 26 opposed leaf pairs with 1–cm leaf width at isocenter was initially employed. Subsequent plans were delivered with a Varian 120 leaf Millennium MLC. The MLC computer controls leaf position as a function of fractional MUs delivered according to a DMLC prescription file. Delivery of IMRT fields is by a step and shoot technique, consisting of a maximum of 15 segments per field.

Using the knowledge gained from the mock treatment of the anthropomorphic phantom, treatment plans were generated for patients according to the in‐house protocol. Clinical planning begins with the patient placed in an immobilization shell equipped with fiducial CT markers to mark a reference point. The patient is scanned and the 3D data set is imported to the TPS. ITP calculations are performed iteratively until an acceptable plan is generated. The field is then exported to a Helax format MLM file which is converted to a Varian format DMLC step and shoot file. This file is then transferred via network to the MLC control computer. The files are then checked with a vendor supplied software package (Shaper, Varian Associates), which ascertains the deliverability of the planned leaf motions (e.g., leaf travel not exceeding allowable physical limits, no carriage irradiation). Individual MLC segments may be manually adjusted as needed. After verifying that measured and planned dose distributions match for the individual BEV fields, the DMLC controller files are then moved to a Varis MLC directory and attached to the Varis patient treatment record.

The verification begins by importing the planned patient IMRT fields into a plan based on a cubic water phantom. All fields are individually delivered and measured using radiographic films. These films are then scanned for comparison to calculated values. Literature in this area is growing, giving credence to the use of film in IMRT QA.^12^ ^,^ ^20^ Calibration films are produced by exposing Kodak XV2 film (Eastman Kodak, Rochester, NY) to $5\times 5{\text{cm}}^{2}$ fields at 10 cm depth in Solid Water™, 90 cm SSD, for 100, 80, 60, 40, 20, 10, 5, and 0 MUs. A calibrated Vidar film scanner (VXR16, Vidar Medical Imaging, Herndon, VA), which can rapidly produce bitmap images, was used to scan the films. An internal recalibration procedure was performed frequently since scanner response seemed to drift, possibly due to change the unit heating up with time. Scanned calibration films are used to generate pixel value to dose H&D curves using a commercial film processing package (PIPS, Masthead Imaging, Nanaimo, BC). This H&D curve is then exported to a file. Bitmap images of the scanned measurement films and the H&D curve data are read by an in‐house program which runs in a commercial math package (Matlab, The Math Works, Natick, MA). The bitmap is re‐sampled from 75 dpi to a 1 mm resolution, the same spatial resolution as the dose calculations, and the H&D curve data is used to convert the measured dose bitmap images to absolute dose. This process allows bitmap images from the scanner to be converted to absolute dose distributions, as well as to convert them into a useful format for comparisons to planned distributions.

TPS BEV dose distributions were calculated for each field at 10 cm depth, 90 cm SSD, at a resolution of 1 mm. The IMRT field is normalized to a $5\times 5{\text{cm}}^{2}$ field of known MU setting. Calculated dose distributions were then exported in RTOG format from the TPS. Measured BEV dose distributions are obtained by exposing films under the same conditions, namely with each film at 10 cm depth with Solid Water buildup, 90 cm SSD. Field alignment was performed by Fourier correlation, found by taking the product of the Fourier transform of one function *a*(*x*) and the Fourier transform of the complex conjugate of the other function *b*(*x*), as in

Corr[a(x),b(x)]=A(k)B*(k).

Correlation is determined between the binary image outlines of the calculated and measured distributions. The outline of the region of interest is computed using the maximum local first derivative, found using the Canny method.^21^ Relative shifts in image alignment were determined between these outlines using Fourier correlation. The shift of the peak of the power spectrum corresponds to the relative shift between the two images. It may be noted that this technique assumes there are no systemic shifts in the delivery (i.e., no constant leaf offsets). No such shifts were noted in the commissioning, and are they are routinely checked for after the leaf sequence has generated by comparing the segment shapes for delivery to the TPS and by checking the portal images on the first day of treatment.

Once the images are registered, the region of interest (ROI) and absolute dose values (calculated and measured) are subtracted from one another. These local differences in absolute dose are used to generate a differential dose error histogram. The physician may then review the dose differences and decide whether or not they are acceptable.

Independent MU checks for IMRT fields have been discussed elsewhere.^22^ ^,^ ^23^ Verification of the MUs calculated by our TPS was achieved by comparing the predicted dose (scaled using a reference field in the plan) to the measured dose from the BEV deliveries. As well, a calculation is performed using a scatter summation method to perform a rough check on the MUs required for each field in order to deliver the planned dose to isocenter for that field.

Additional testing of the individual fields consisted of looking at IMRT sub fields (static segments) by exporting them as individual MLC shapes and testing the modeling of their relative distributions and dose factors. A small volume chamber was also used to measure the output for individual IMRT fields in low gradient regions. The results are compared to calculated values.

**Table I:** Distribution of CR portal images studied by anatomical sites.

Field size (cm×cm)	16×16	10×10	6×6	3×4	2×4	2×3	1×3
Helax	1.077	1.000	0.918	0.854	0.836	0.827	0.771
Film	1.075	1.000	0.918	0.824	0.805	0.775	0.735
Ion chamber	1.069	1.000	0.919	0.845	0.817	0.808	0.733

The summed result of all seven fields was checked in the anthropomorphic phantom. Both transverse films and TLDs were loaded into the phantom and used to verify the delivery of planned point doses and the overall dose distribution. The TLDs used were $0.3\times 0.3\times 0.1{\text{cm}}^{3}$ LiF chips, and were calibrated and read using an automated TLD reader. The film used was Kodak XV2 that was sized and cut in a darkroom and light packed using opaque tape.

Routine IMRT delivery QA consisted of using an in‐house routine to print all leaf segments for each DMLC field, along with a rough estimate of the net energy fluence from each field (much like the “simulated film” of the MLC Shaper program). This allows therapist verification of leaf shape during delivery. Therapists also perform a routine morning check, and a dynamic delivery report of this is reviewed for any anomalous values.

Other steps in the IMRT QA process include performing a positioning check on the MLC in dynamic mode. This is currently achieved through the delivery of a test DMLC file on a radiographic film, and the dose dynamic log is also checked for abnormalities. Current clinical QA consists of checking the first and last leaf segments using overheads placed on the accessory tray. Segments are also checked visually on MLC workstation during delivery.

## RESULTS

Comparison of relative dose factor measurements using film and ion chamber and those predicted by the TPS for various MLC defined fields reveals minimal variations for all but the smallest fields (see Table I), where discrepancies were of the order of 2–3.5%. Given the limited amount of time spent delivering such very small fields, and given that they are used only to augment doses to small regions, this variation was found to be quite acceptable.

**Figure 2:**
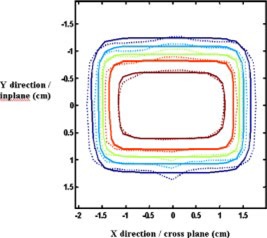
(Color) Small field distribution, measured and calculated. Leakage through the closed leaves below the *Y* jaws is visible around the X=0 position.

Planned versus measured field distributions for small fields centered about the CAX were found to be in good agreement (see Fig. 2). The same field shape was delivered as an off axis field CAX, with similar good agreement observed.

**Figure 3:**
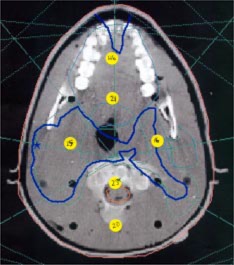
(Color) Placement of the verification TLDs within the anthropomorphic phantom.

All seven fields of the anthropomorphic phantom IMRT plan were delivered, and the results were measured with the TLDs (see Fig. 3) and planar film. The results for the TLDs are summarized in Table II. Good agreement between the TLDs and the calculated values was observed, with most errors under 5%, fewer in the 5–10 % region, along with a spurious reading over 10%. Given the typical errors associated with TLDs, these variations were mostly within the expected range (i.e., 5%). Where the results are in the 5–10% range, these were typically in low dose regions, where small absolute dose differences can lead to large percentage errors, and are likely due to small inaccuracies in the TPS modeling of the leaf transmission and penumbral region. The one TLD with >10% error returned a dose of zero in what was a misread by the automated reader. The planar film had too many artifacts to be of much use.

**Figure 4:**
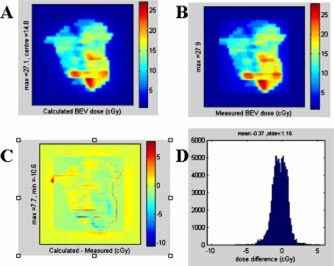
(Color) BEV of clinical IMRT field, calculated (A) and measured (B). These are used to generate a difference map (calculated‐measured) (C), as well as a histogram of number of pixels vs absolute dose difference between calculated and measured (D).

A comparison of the BEV film measurement and calculation was performed for each patient treated, as well as for the mock delivery on the anthropomorphic phantom. Figure 4 shows the results for an IMRT field from the initial clinical plan for patient number 1. The BEV data was used to generate a dose difference map [Fig. 4(C)], as well as a differential dose error histograms [Fig. 4(D)]. The dose difference map yields a comparison that gives a sense of the area involved in a discrepancy and the differential dose error histograms gives a good representation of the spread of these errors. Tests of all eight fields from a representative clinical IMRT plan show some discrepancies between the planned and delivered doses for the BEV calculations, as detailed in Table III.

An example of the results from the BEV comparison of a clinical field is given in Table III. The results are generally very good. The measured doses had low mean dose deviations (typically of the order of less than 1.5 cGy). As well the standard local error was only of the order of 2 cGy. The deviation between the planned and measured maximum dose points is generally less than 2%.

**Table II:** Difference in dose between measured (TLD) and calculated values for Rando plan

(Measured‐calculated) ICRU dose			
% difference	<5%	5–10%	>10%
# TLDs	17	8	1

It is thought that the 5% discrepancy was due to the dosing due to transmission through the closed leaf end. Planning and delivery of DMLC that allows for carriage motion may eventually mitigate this effect. The one case of the 16.5% difference was caused by atypical leaf sequencing resulting in a series of adjacent single leaf openings. The calculated modeling of these one cm wide fields is known to have some inaccuracies. This sequencing issue has since been resolved by the latest software release for the TPS.

Several difficulties were encountered in the delivery of the planned IMRT fields. These were rooted in mechanical limitations and characteristics of the MLC. The inherent limitations of the MLC that posed problems were transmission through the “closed” leaf end of the MLC and the tongue and groove problem. Difficulties were also encountered with the delivery of large IMRT fields due to leaf travel restrictions. These leaf travel limitations are imposed on fields over a certain size due to (1) possible carriage irradiation, (2) leaf over travel restrictions, as well as (3) restrictions on allowable leaf separation.

**Figure 5:**
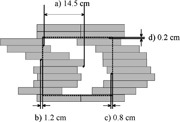
The allowed and assumed positions of leaves and jaws relative to each other. The maximum separations between any two leaves is 14.5 cm (shown in a). This is a consequence (b) the closest point the MLC assembly may approach the jaw edge and (c) the jaw setting being relative to the maximum leaf position in any DMLC treatment. The lateral jaws are set at 0.2 cm greater than the edge of the unused leaves.

There was a difficulty encountered in the delivery of certain fields over 14.5 cm wide (in the leaf travel direction) that was due to transmission between the closed leaves. The maximum travel distance between any two leaves on the same side of the carriage is 14.5 cm during the delivery of a field [separation “a” in Fig. 5(a)]. While there generally was not a problem with the moving

**Table III:** Gauges of the difference between the measured and calculated values for a clinical IMRT plan

Field #	Mean dose deviation (cGy)	Std. dose deviation (cGy)	Max. calculated dose (cGy)	Max. measured dose (cGy)	% difference
1	–1.04	3.11	78.3	78.5	–0.3
2	–1.61	1.94	45.6	47.8	–4.8
3	–0.95	2.00	53.3	53.3	0.0
4	–0.84	2.21	69.2	67.9	1.9
5	–0.76	2.39	93.8	91.7	2.2
6	–1.30	1.87	55.7	56.6	–1.6
7	–4.26	3.21	64.7	75.4	–16.5
8	–1.33	2.40	77.2	78.1	–1.2

leaves for smaller fields, the TPS algorithm only accounts for the over travel issue on a per segment basis. This means, for example, that no single segment of the 15 segments in a DMLC delivery will have any two leaves on the same side of the carriage more than 14.5 cm apart. This restriction is not applied between this segment and the other segments, which may result in having segments as shown in Figs. 6(a) and Fig. 6(c). The segment shown as Figure 6(c) must be manually modified to that shown in Fig. 6(b). As well, the closed leaves in the field can be a problem; unused leaves are simply designated as –999 in the MLM file outside the range of the moving leaves, and inside they are set to close at a constant position. The result of this is twofold: (i) unused leaves may not be moved off axis [see Fig. 6(d)], and (ii) if unused leaf positions are set to a constant value, it results in a high level of leakage through the closed leaf ends (as shown in Fig. 6).

**Figure 6:**
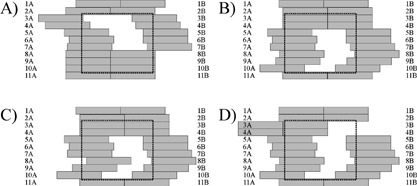
Example segments of a step and shoot delivery. The jaw positions for a two segment delivery using the fields shown in (A) and (B) are determined by leaves 3A and 8B in fields 1 and 2 respectively. Since the TPS does not restrict intersegment over travel, the situation shown in (C) may arise where the second segment could have an undeliverable leaf position. The second segment is shown in (D) with the closed leaves moved off axis, creating an undeliverable segment.

Due to this limitation, a solution had to be found to deliver larger fields. Three solutions were explored. One is to not deliver a single field larger than 14.5 cm, but rather deliver two smaller fields from the same gantry angle to cover a greater width. Another solution is to step the closed leaf ends across the maximum distance allowed in the field (from *start to stop*) as a function of fractional MUs that are to be delivered at the end of that segment (ΔI) as per:

$$\text{leaf}{\left(x\right)}_{i}=\text{start}+(\Delta I)\cdot \frac{\left(\text{start‐stop}\right)}{I_{\text{total}}},$$

where the stating and stopping positions are defined as follows:

$$\text{start}=14.5+{\text{maxleaf}}_{A},\;\;\;\text{stop}=14.5-{\text{maxleaf}}_{B},$$

where ${\text{maxleaf}}_{A}$ and maxleaf^*B*^ are the maximum leaf positions for each bank of leaves throughout the entire treatment. We referred to this method as static feathering of the closed leaves. The third solution is to try and limit, with the use of a collimator rotation, the projected BEV width of the field in order to circumvent this situation entirely.

**Figure 7:**
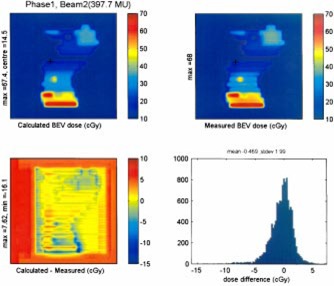
(Color) An example of IMRT beam verification using difference map and local difference error histogram which highlights a suboptimal leaf sequencing for the step and shoot, resulting in an obvious tongue and groove problem.

Tongue and groove problems were visible on the dose verification films as well (see Fig. 7). These are well known^24^ to arise from the mechanical design of the MLC, but may be mitigated by the leaf sequencing algorithm used.^25^ Since leaf sequencing is handled internally by our TPS, and given that the effects are not seen to pose a large perturbation on the delivery, tongue and groove effects are currently accepted as a minor delivery shortcoming.

To date, several IMRT treatments have been delivered. The overall results of all treatments (to mid 2001) are summarized in Table IV. There have been ten patient treatments that have been planned, verified and finished treatment, including four patients who have had boosts. Of these deliveries, two used 6 fields, five used 7 fields, and four used 8 fields. The treatment was to deliver 50 Gy median dose to the PTV. For the first phase treatments, there have been an average of 12 segments per MLM field, with a total number of MUs ranging from 944 to 1645 (average of 1266, giving a modulation factor of 2.5, assuming 500 MUs conventionally). The average mean dose difference has been –1.1 cGy, or about –2% of max dose. The average standard deviation of differences has been 2.8 cGy, or about 4% of max dose. For the two boost plans, one used 6 fields and one used 8 fields. The total MUs delivered were 780 and 802, respectively, giving a modulation factor of 1.6. The average mean dose difference was – 1.2 cGy, or about –2% of maximum field dose. The average standard deviation of differences was 2.4 cGy, or about 4% of maximum field dose.

**Table IV:** Summary of results from QA measurements of the agreement between the measured and calculated doses for all clinical IMRT plans to date.

Patient #	Number of fields	Minimum/Maximum mean dose deviation for all fields (cGy)	Average of mean dose deviation for all fields (cGy)	Minimum/Maximum mean dose deviation for all fields (cGy)	Average of std. dose deviation for all fields (cGy)
1 (Phase1)	8	–1.9,0.7	–0.9	1.4, 2.7	2.0
1 (Phase2)	5	–4.6,–1.8	–1.6	1.4, 4.8	2.4
2	8	‐1.7,‐0.6	–1.0	1.5, 3.5	2.2
3	6	‐1.3,1.2	–0.3	2.2, 6.0	3.9
4	7	‐3.4,‐0.4	–1.8	1.3, 4.1	2.4
5 (Phase1)	8	–2.3,‐0.2	–0.8	1.7, 3.9	2.7
5 (Phase2)	8	–3.0,0.0	–0.8	1.4, 3.7	2.4
6	8	–1.5,3.0	–0.2	2.3, 3.9	3.3
7	8	‐4.3,‐0.8	–1.5	1.9, 3.2	2.4

The delivery of the clinical fields required, initially, roughly 28 min from the time the patient enters the treatment vault to the time they leave. This time has since been reduced to about 15 min which is the length of a standard treatment slot at this institution. This reduction in treatment times has been aided by increased comfort and familiarity on the part of the radiation therapists, as well as the use of an automated set‐up function from our “record and verify” system.

## DISCUSSION

Our initial experience has made it clear that there is a need for more training for dosimetrists, physicists, and oncologists who will be involved in the IMRT process, such that everyone will gain a greater comfort with the technique. From our initial experience with the ITP process over the last year, it has become apparent that a number of TPS tools are needed to streamline the IMRT and ITP process in the clinic. On the TPS side, such tools would include user options for making it easier to introduce a user defined number of segments and levels to be used. Allowing the user to specify the minimum field size to be used in any MLC segment (e.g., $3\times 3{\text{cm}}^{2}$) would preclude any discomfort in the modeling of excessively small MLC segments (e.g., $1\times 0.5{\text{cm}}^{2}$). In‐house tools which are being developed include better dose agreement metrics, such as distance to agreement and a gamma index,^26^ as well, a new film for dosimetry is being investigated (EDR2, Eastman Kodak, Rochester, NY). EPID dose verification would greatly decrease the time currently spent on film verification.

A number changes are also desired with respect to the MLC handling within the TPS. These would include better handling of the allowed leaf travel within any given field. Currently, TMS does restrict leaf travel to under 14.5 cm, but only within any given segment. This means that the overall span between leaves can exceed 14.5 cm within an MLM treatment which, in our case, consists of 15 segments. The MLC is not precisely modeled in terms of leaf travel and collimation around the MLC aperture. This is due in part to the differing method in which the MLC is integrated in Varian units, it is a tertiary add on, below two sets of collimating jaws, as opposed to Siemens or Elekta machines, where the MLC is an integrated part of the collimation. As well, Varian does not yet have support for carriage movement for its DMLC, something that is currently assumed in the Helax TMS handling of the DMLC.

**Figure 8:**
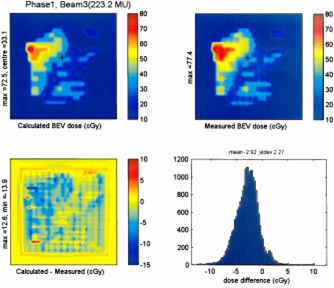
(Color) An example of IMRT beam verification for a large field which shows a streaking due to stepping the closed leaves through the field, resulting in streaks of higher dose in the delivery.

The current work around for the leaf travel issue is chiefly to limit field sizes to under 14.5 cm in the leaf travel direction by choosing a collimator rotation which limits the width in the leaf travel direction (gantry angles are approximately equally spaced about the patient). If fields sizes must be over 14.5 cm, it would be beneficial to allow the user to specify how closed leaves should be junctioned (e.g., statically or dynamically moved with each segment), or to allow more seamless junctioning of split fields, as others have done.^27^ We are still evaluating which method is the best, but currently employ static feathering for some large field segments, and occasionally create two smaller fields for a single gantry angle. The effect of the static feathering can be seen on the verification films (see Fig. 8).

**Figure 9:**
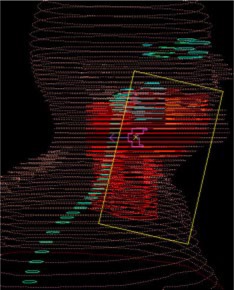
(Color) An example of planner assistance in achieving the goals of the inverse plan by employing collimator rotation to facilitate sparing of an organ at risk on an IMRT field (spinal cord, shown in green).

Collimator rotations and jaw positions have been employed in some fields to force sparing of certain organs at risk (e.g., Fig. 9). This sparing is employed for some fields in order to more readily obtain an acceptable solution from the ITP software.

We anticipate the use of EPID verification instead of film will simplify the verification phase of the QA. TLD or gel dose verification may be used at a future date to perform a 3D error analysis, and to investigate 3D combination of errors.

## CONCLUSIONS

A series of tests were devised to test the reliability of the calculation and delivery of IMRT fields using an ITP module on a commercial TPS. The testing centered on both the general questions of MLC modeling for small fields, as well as specific calculation accuracy of clinical

IMRT fields. The result of our tests on the MLC modeling on our TPS indicates a small problem which seems to exist with the calculated relative dose factor of small MLC fields, which is on the order of 2–3 % for the smallest fields.

Custom software is currently required to verify, modify, and transfer the Helax TMS generated MLM data to Varis for delivery on a Varian linac. The results from the various BEV measurements for the individual fields imported to a flat phantom test case indicate good agreement between the planned and delivered. The results of the films and TLDs for the Solid Water™ phantom indicate good agreement between planned and delivered doses.

To‐date, Helax‐TMS 5.1 has been used to inverse‐plan, verify, and treat ten Nasopharynx patients sparing one or both parotid glands. The initial observations have been encouraging; the results of the BEV tests indicated generally good agreement between planned distribution and dose levels. We are seeing an average mean difference of 1.1 cGy, or 2% of the maximum planned dose, and an average standard deviation of differences of 2.8 cGy, or 4% of maximum planned dose.

The initial time commitment of the IMRT QA process has been significant, but the process is becoming streamlined. We currently commit about 8 h of verification time per plan. As we gain more familiarity with the planning and delivery of these IMRT fields, it is expected that this process will evolve and be streamlined, and the time required per patient will drop.

Our early experience has led us to try to use field sizes less than 14.5 cm where possible to allow closing leaves outside field to circumvent leakage through the closed leaf ends. This avoids rather than solves the in‐field closed leaf leakage problem, which may yet be addressed in some other fashion. One possibility is the enabling of jaw motion for each segment in a field. The closed leaf position has not been moved dynamically as it was feared this may give rise to a larger separation than with the static stepping, as well as increasing wear on the MLC assembly (the so‐called “chattering teeth” problem).

It is also hoped that future releases of the TPS will include tools to aid in the IMRT QA process. These tools would assist with the handling of the closed leaf issues as well as greater ease in the copying of the MLM beams to a water phantom plan to aid in verification. As well, the arrival of Dicom‐RT plan import in TMS v6.0 and Varis's Generation 6.0 Dicom‐RT support should ease the export of MLM plans.

## ACKNOWLEDGMENTS

The authors would like to thank Gary Morisson and Ken Hennig for their help in manufacturing the IMRT phantoms. We would also like to thank Heather Thompson of the Medical Physics Department for help with the TLD measurements. Thanks to Dr. Mathew Parliament and Dr. Elizabeth Kurien of Radiation Oncology for establishing the in‐house protocol. Lori Underwood RTT (dosimetry), Phil Kelly RTT, and Denis Weber (therapists) provided invaluable assistance in the planning and delivery of the IMRT plans. Many thanks also go to Anders Gustafsson and Anders Murman of Helax AB (MDS Nordion) for technical advice. The financial support and provision of pilot software by Helax AB (MDS Nordion) is gratefully acknowledged.

## References

[1] R. C. Urtasun , “Does improved depth dose characteristics and treatment planning correlate with a gain in therapeutic results?” Int. J. Radiat. Oncol., Biol., Phys. 22, 235–239 (1992). 1740390 10.1016/0360-3016(92)90038-j

[2] P. Harari and T. Kinsella , “Advances in radiation therapy for head and neck cancer,” Curr. Opin. Oncol. 7, 248–54 (1995). 7654827 10.1097/00001622-199505000-00010

[3] A. Eisbruch , I. A. Ship , and M. K. Martel , “Parotid gland sparing in patients undergoing bilateral head and neck irradiation: Techniques and early results,” Int. J. Radiat. Oncol., Biol., Phys. 36, 469–480 (1996). 8892473 10.1016/s0360-3016(96)00264-7

[4] A. Eisbruch , L. H. Marsh , and M. K. Martel , “Comprehensive irradiation of head and neck cancer using conformal multisegmental fields: Assessment of target coverage and noninvolved tissue sparing,” Int. J. Radiat. Oncol., Biol., Phys. 41, 559–568 (1998). 9635702 10.1016/s0360-3016(98)00082-0

[5] A. Eisbruch , R. K. Ten Haken , and M. K. Hyungjin , “Dose, volume, and function in parotid salivary glands following conformal and intensity modulated irradiation of head and neck cancer,” Int. J. Radiat. Oncol., Biol., Phys. 45, 577–587 (1999). 10524409 10.1016/s0360-3016(99)00247-3

[6] D. Low , C. K. S. Chao , S. Mutic , R. L. Gerber , C. A. Perez , and J. A. Purdy , “Quality assurance of serial tomotherapy for head and neck patient treatments,” Int. J. Radiat. Oncol., Biol., Phys. 42, 681–692 (1998). 9806530 10.1016/s0360-3016(98)00273-9

[7] J.‐S. Tsai , D. E. Wazer , M. N. Ling , J. K. Wu , M. Fagundes , T. DiPetrillo , B. Kramer , M. Koistiner , and M. J. Engler , “Dosimetric verification of the dynamic intensity modulated therapy of 92 patients,” Int. J. Radiat. Oncol., Biol., Phys. 40, 1213–1230 (1998). 9539579 10.1016/s0360-3016(98)00009-1

[8] D. Verellen , N. Linthout , D. Van Den Berge , A. Bel , and G. Storme , “Initial experience with intensity‐modulated conformal radiation therapy for treatment of the head and neck region,” Int. J. Radiat. Oncol., Biol., Phys. 39, 99–114 (1997). 9300745 10.1016/s0360-3016(97)00304-0

[9] D. A. Low , R. L. Gerber , S. Mutic , and J. A. Purdy , “Phantoms for IMRT dose distribution measurement and treatment verification,” Int. J. Radiat. Oncol., Biol., Phys. 40, 1231–1235 (1998). 9539580 10.1016/s0360-3016(97)00910-3

[10] T. Knoos , C. Ceberg , L. Weber , and P. Nilsson , “The dosimetric verification of a pencil beam based treatment planning system,” Phys. Med. Biol. 39, 1609–1628 (1994). 15551534 10.1088/0031-9155/39/10/007

[11] Lei Xing , Bruce Curran , Robert Hill , Tim Holmes , Lijun Ma , Kenneth M. Forster , and Arthur L. Boyer , “Dosimetric verification of a commercial inverse treatment planning system,” Phys. Med. Biol. 44, 463–478 (1999). 10070795 10.1088/0031-9155/44/2/013

[12] C. Burman *et al*, “Planning, delivery, and quality assurance of intensity modulated radiotherapy using dynamic multileaf collimator: Astrategy for large scale implementation for the treatment of carcinoma of the prostate,” Int. J. Radiat. Oncol., Biol., Phys. 39, 863–873 (1997). 9369136 10.1016/s0360-3016(97)00458-6

[13] Radiation Therapy and Oncology Group , RTOG Protocol H‐0022: Conformal and intensity modulated irradiation for oropharyngeal cancer, Philadelphia, PA, 2000 (available at: http://www.rtog.org/members/protocols/h00221)

[14] Anders Ahnesjo , Mikael Saxner , and Avo Trepp , “A pencil beam model for photon dose calculation,” Med. Phys. 19, 263–273 (1992). 1584117 10.1118/1.596856

[15] Anders Ahnesjo , Tommy Knoos , and Anders Montelius , “Application of the convolution method for calculation of output factors for therapy photon beams,” Med. Phys. 19, 295–301 (1992). 1584120 10.1118/1.596859

[16] Anders Ahnesjo , “Analytic modeling of photon scatter from flattening filters in photon therapy beams,” Med. Phys. 21, 1227–1235 (1994). 7799864 10.1118/1.597205

[17] Anders Ahnesjo , “Collimator scatter in photon therapy beams,” Med. Phys. 22, 267–278 (1995). 7596315 10.1118/1.597450

[18] Anders Ahnesjo , Lars Weber , and Per Nilsson , “Modeling transmission and scatter for photon beam attenuators,” Med. Phys. 22, 1711–1720 (1995). 8587523 10.1118/1.597534

[19] R. H. Byrd , P. Lu , J. Nocedal , and C. Zhu , “A limited memory algorithm for bound constrained optimization,” Sci. Comput. (USA) 5, 16–23 (1995).

[20] C. Danciu , B. Proimos , J.‐C. Rpsewald , and B. Mijnheer , “Variation of sensitometric curves of radiographic films in high energy photon beams,” Med. Phys. 28, 966–974 (2001). 11439493 10.1118/1.1376443

[21] J. F. Canny , “A computational approach to edge detection,” IEEE Trans. Pattern Anal. Mach. Intell. 8, 679–698 (1986). 21869365

[22] A. Boyer , L. Xing , C‐M. Ma , B. Curran , R. Hill , A. Kania , and A. Bleier , “Theoretical considerations of monitor unit calculations for intensity modulated beam treatment planning,” Med. Phys. 26, 187–195 (1999). 10076972 10.1118/1.598502

[23] L. Xing , Y. Chen , G. Luxton , J. G. Li , and A. L. Boyer , “Monitor unit calculation for an intensity modulated photon field by a simple scatter‐summation algorithm,” Phys. Med. Biol. 45, N1–N7 (2000). 10730973 10.1088/0031-9155/45/3/401

[24] X. Wang , S. Spirou , T. LoSasso , C. Chui , and R. Mohan , “Dosimetric verification of an intensity modulated treatment,” Med. Phys. 23, 317–327 (1996). 8815373 10.1118/1.597661

[25] J. van Santvoort and B. Heijmen , “Dynamic multileaf collimation without ‘tongue‐and‐groove’ underdosage effects,” Phys. Med. Biol. 41, 2091–105 (1996). 8912383 10.1088/0031-9155/41/10/017

[26] W. Harms , D. Low , J. Wong , and J. Purdy , “A software tool for the quantitative analysis of 3D dose calculation algorithms,” Med. Phys. 25, 1830–1836 (1998). 9800688 10.1118/1.598363

[27] Qiuwen Wu , Mark Arnfield , Shidong Tong , Yan Wu , and Radhe Mohan , “Dynamic splitting of large intensity‐modulated fields,” Phys. Med. Biol. 45, 1731–1740 (2000). 10943915 10.1088/0031-9155/45/7/302

